# Obesidade e Risco de Hipertensão: Um Problema Crescente em Crianças e Adolescentes

**DOI:** 10.36660/abc.20220940

**Published:** 2023-02-16

**Authors:** José Geraldo Mill

**Affiliations:** 1 Hospital Universitário Cassiano Antônio Moraes Vitória ES Brasil Hospital Universitário Cassiano Antônio Moraes, Vitória, ES – Brasil; 2 Departamento de Ciências Fisiológicas Universidade Federal do Espírito Santo Vitória ES Brasil Departamento de Ciências Fisiológicas – Universidade Federal do Espírito Santo, Vitória, ES – Brasil

**Keywords:** Hipertensão/genética, Obesidade, Criança, Estilo de Vida, Adiposidade, Adolescente, Exercício

A hipertensão arterial (HA) afeta cerca de 30% dos adultos no Brasil.^1^Condição anteriormente rara em crianças e adolescentes, esse quadro vem se modificando dada a pandemia de obesidade ocorrendo atualmente nesta faixa etária.

A HA primária é uma doença complexa pois seu aparecimento depende de predisposição genética e de fatores ligados ao estilo de vida.^2^Não existe ainda um escore genético robusto e de fácil obtenção para ser usado clinicamente como preditor de HA.^3^ O mesmo não ocorre com os fatores ligados ao estilo de vida, pois sabe-se que sedentarismo, obesidade, consumo elevado de sal e resistência à insulina predispõem ao aumento da pressão ao longo da vida.

**Figura 1:**
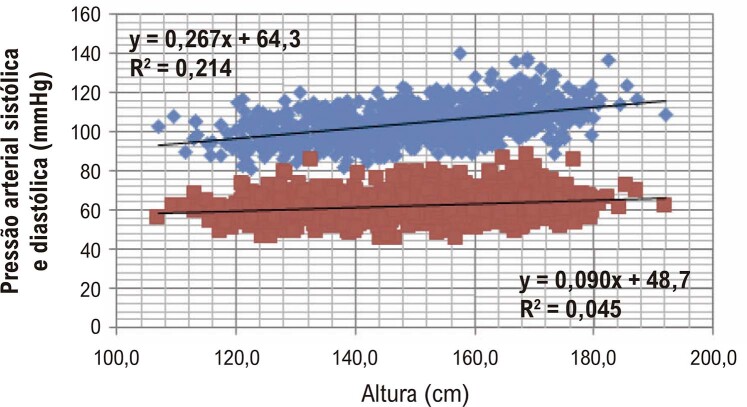
– Pressão Arterial em crianças e adolescentes de 6-17 anos (meninos: 478; meninas: 373). Estação Conhecimento, Serra, ES (2014-2016).

Em recém-nascidos, a pressão arterial (PA) sistólica situa-se entre 70 e 90 mmHg e a diastólica entre 40 a 60 mmHg.^4^ A expansão da rede vascular com o crescimento, e a consequente perda de energia da circulação com o atrito e aumento da resistência vascular periférica, exigem aumento do gradiente pressórico entre o coração e a microcirculação para que a pressão de filtração nos capilares, determinada pelas forças de Starling, seja mantida constante. Assim, há necessidade de aumento progressivo da pressão nas grandes artérias com o crescimento ponderal que, em crianças e adolescentes, se correlaciona com a idade (Figura 1).^5^

Assim, a definição de PA normal até 18 anos é feita em função de percentis, e não em valor fixo como no adulto e, portanto, tabelas específicas devem ser consultadas para avaliação pressórica. Em crianças e adolescentes a ‘pressão alta’ decorre da acentuação do incremento pressórico com a idade, a qual pode ocorrer por causas identificáveis (doenças renais, tumores, etc.), quando se obtém o diagnóstico de ‘HA secundária’ ou sem causas definidas, tal como ocorre na HA essencial do adulto.

O acúmulo de gordura é o principal fator que acentua o incremento pressórico durante o crescimento, sendo o principal fator que predispõe ao aparecimento da HA na infância e adolescência.^6^ A pandemia de obesidade, afetando fortemente crianças e adolescentes, traz duas consequências importantes: aumento da resistência à insulina (predispondo ao diabetes tipo 2) e da PA.^7^ Em estudo de abrangência nacional (ERICA) no qual a PA foi medida em mais de 70 mil escolares de 12 a 17 anos, a prevalência de HA alcançou a cifra de 9,6% (IC 95%: 9,0-10,3%).^5^

Poucos estudos, entretanto, foram realizados no Brasil avaliando a incidência de HA. Este número dos Arquivos Brasileiros de Cardiologia traz artigo com relato de seguimento de 3 anos em 469 escolares de 7-17 anos em cidade do Rio Grande do Sul. Observou-se incidência de HA de 11,5%. Dentre os vários preditores incluídos no modelo analítico, incluindo um polimorfismo genético, constatou-se que, de longe, o excesso de peso foi o principal, com medida de associação (odds ratio = 4,84; IC 95% = 1,57-14,95) muito elevada.^6^Mas outro dado relevante no estudo foi a grande frequência de mudança de classificação pressórica (normotensão, pressão elevada e hipertensão) dentro dos grupos ao se comparar as medidas pressóricas iniciais e as do seguimento. Assim, dos 16 identificados como portadores de ‘hipertensão sistólica’ na avaliação inicial, apenas 7 mantiveram essa classificação na reavaliação de 3 anos. Entre os 18 classificados como portadores de ‘pressão arterial elevada’ na primeira avaliação, 6 migraram para o grupo de hipertensão, 2 permaneceram no mesmo grupo e 10 migraram para normotensão.

Esses dados sinalizam que o diagnóstico de ‘hipertensão’ deve ser feito com grande cautela em crianças e adolescentes, sendo necessárias três aferições separadas por, pelo menos, uma semana. Deve-se dar preferência ao uso de aparelho oscilométrico com braçadeira adequada à circunferência do braço. Crianças e adolescentes são mais susceptíveis ao efeito do jaleco branco, exigindo cuidados adicionais na interpretação das medidas pressóricas, principalmente obtidas fora do ambiente clínico, como em escolas.^7^

Os dados indicam a necessidade de vigilância pressórica de crianças e adolescentes, principalmente em presença de sobrepeso ou obesidade e que medidas de prevenção devem ser intensificadas com mais rigor nesse grupo. Duas intervenções são mandatórias: redução do consumo de produtos industrializados pois contém teores mais elevados de sódio e carbo-hidratos simples (frutose e sacarose) e aumento do gasto calórico em atividades ou exercícios físicos. Obesos também apresentam maior ingestão de sal, mesmo após ajuste para o consumo calórico.^8^ Valores pressóricos elevados na fase de crescimento contribuem para a remodelação vascular e aumento da rigidez arterial, predispondo à HA no adulto.^9^ O exercício físico seria a medida mais efetiva para frear o crescimento da obesidade e, consequentemente, melhorar a sensibilidade à insulina e atenuar o incremento pressórico com a idade. Nesta fase da vida o exercício deve constituir atividade lúdica e não um hábito de vida, como no adulto. Essas atividades devem ocorrer no lazer e na escola. Ensaios clínicos mostram que o aumento do número de aulas de educação física atenua o incremento pressórico com a idade,^10^ prevenindo o desenvolvimento da HA.
