# Supplementary material for: Obesidade e Risco de Hipertensão: Um Problema Crescente em Crianças e Adolescentes
Source: Arq Bras Cardiol. 2023 Feb 16;120(2):e20220940. [Article in Portuguese] doi: 10.36660/abc.20220940 (PMC12080618; doi:10.36660/abc.20220940)
Supplement: Translation

# Translation: Obesity and Risk of Hypertension: A Growing Problem in Children and Adolescents

**Authors:** José Geraldo Mill

**Affiliations:** 1 Brasil Hospital Universitário Cassiano Antônio Moraes, Vitória, ES – Brasil; 2 Brasil Departamento de Ciências Fisiológicas – Universidade Federal do Espírito Santo, Vitória, ES – Brasil

**Keywords:** Hypertension/genetic, Obesity, Child, Lifestyle, Adiposity, Adolescent, Exercise

Arterial hypertension (AH) affects approximately 30% of adults in Brazil.^1^ A previously rare condition in children and adolescents, this situation has been changing given the obesity pandemic currently occurring in this age group.

Primary AH is a complex disease, as its appearance depends on genetic predisposition and factors linked to lifestyle.^2^ There is still no robust and easily obtainable genetic score to be used clinically as a predictor of AH.^3^ The same does not occur with the lifestyle factors, as it is known that a sedentary lifestyle, obesity, high salt intake and insulin resistance, predisposes to blood pressure increase throughout life.

In newborns, systolic blood pressure (BP) is between 70 and 90 mmHg and diastolic between 40 and 60 mmHg.^4^ The expansion of the vascular network with growth, and the consequent loss of energy by the circulation with friction and increase in peripheral vascular resistance, require an increase in the pressure gradient between the heart and the microcirculation so that the filtration pressure in the capillaries, determined by Starling forces, is kept constant. Thus, there is a need for a progressive increase in pressure in the great arteries with height weight growth, which correlates with age in children and adolescents (Figure 1).^5^

**Figure 1:** – Blood Pressure in children and adolescents aged 6-17 years (boys: 478; girls: 373). Estação Conhecimento, Serra, ES (2014-2016).

Thus, the definition of normal BP up to 18 years of age is based on percentiles and not a fixed value as in adults; therefore, specific tables must be consulted for blood pressure assessment. In children and adolescents, ‘high blood pressure’ results from the increase in blood pressure with age, which can occur due to identifiable causes (kidney diseases, tumors, etc.), when a diagnosis of ‘secondary AH’ is obtained or without defined causes, as occurs in the adult essential AH.

Fat accumulation is the main factor that accentuates the pressure increase during growth, being the main factor that predisposes the appearance of AH in childhood and adolescence.^6^ The obesity pandemic, strongly affecting children and adolescents, has two important consequences: increased insulin resistance (predisposing to type 2 diabetes) and BP.^7^ In a nationwide study (ERICA) in which BP was measured in more than 70,000 schoolchildren aged 12 to 17 years, the prevalence of AH reached 9.6% (95% CI: 9.0-10.3%).^5^

Only some studies, however, were carried out in Brazil evaluating the incidence of AH. This number of the* Arquivos Brasileiros de Cardiologia* publishes as article with a 3-year follow-up of 469 schoolchildren aged 7-17 years in a city in Rio Grande do Sul. There was an incidence of AH of 11.5%. Among the various predictors included in the analytical model, including a genetic polymorphism, it was found that, by far, overweight was the main one, with a measure of association (odds ratio = 4.84; 95% CI = 1.57- 14,95) very high.^6^ But another relevant data in the study was the high frequency of changes in pressure classification (normotension, high blood pressure, and hypertension) within groups when comparing initial and follow-up blood pressure measurements. Thus, of the 16 identified as having ‘systolic hypertension’ in the initial assessment, only 7 maintained this classification at the 3-year reassessment. Among the 18 classified as having ‘high blood pressure’ in the first evaluation, 6 migrated to the hypertension group, 2 remained in the same group, and 10 migrated to normotension.

These data indicate that the diagnosis of ‘hypertension’ must be made with great caution in children and adolescents, requiring three measurements separated by at least one week. Preference should be given to using an oscillometric device with a cuff suitable for the arm’s circumference. Children and adolescents are more susceptible to the effect of the white coat, requiring additional care in the interpretation of blood pressure measurements, mainly obtained outside the clinical environment, such as in schools.^7^

The data indicate the need for blood pressure monitoring in children and adolescents, especially in the presence of overweight or obesity, and that prevention measures should be intensified more rigorously in this group. Two interventions are mandatory: reducing the consumption of industrialized products because they contain higher levels of sodium and simple carbohydrates (fructose and sucrose) and increasing caloric expenditure in activities or physical exercises. Obese individuals also have higher salt intake, even after adjusting for caloric intake.^8^ High blood pressure values in the growth phase contribute to vascular remodeling and increased arterial stiffness, predisposing to AH in adults.^9^ Physical exercise would be the most effective measure to curb the growth of obesity and, consequently, improve insulin sensitivity and attenuate the pressure increase with age. At this stage of life, exercise should be a recreational activity and not a habit of life, as in adults. These activities should take place at leisure and school. Clinical trials show that increasing the number of physical education classes attenuates the pressure increase with age,^10^ preventing the development of AH.
